# Microalgae-Derived Extracellular Vesicle-Loaded 3D Alginate Hydrogels Promote In Vitro Skin and Bone Repair through Dual Fibroblast and Mesenchymal Stem Cell Modulation

**DOI:** 10.1021/acsabm.5c02229

**Published:** 2026-01-03

**Authors:** Noemi De Cesare, Luna Ardondi, Tommaso Pusceddu, Lucia Sileo, Maria Pia Cavaleri, Ilaria Vitali, Francesco Grassi, Brunella Grigolo, Giuseppe Pezzotti, Ugo D’Amora, Letizia Ferroni, Alfredo Ronca, Barbara Zavan

**Affiliations:** † Institute of Polymers, Composites and Biomaterials—National Research Council (IPCB-CNR), Naples 80125, Italy; ‡ Department of Medical Sciences, 9299 University of Ferrara, Ferrara 44121, Italy; § Maria Cecilia Hospital, GVM Care and Research, Cotignola 48033, Italy; ∥ Laboratorio RAMSES, IRCCS Istituto Ortopedico Rizzoli, Bologna 40136, Italy; ⊥ Biomedical Engineering Center, 12880 Kansai Medical University, 1-9-11 Shin-machi, Hirakata, Osaka 573-1191, Japan; # Institute of Polymers, Composites and Biomaterials—National Research Council (IPCB-CNR), Lecco 23900, Italy

**Keywords:** microalgae-derived extracellular vesicles, diabetic ulcer care, sodium alginate, hydroxyapatite, 3D printed hydrogel

## Abstract

Chronic skin wounds with underlying bone exposure represent a major clinical challenge, characterized by impaired healing and limited tissue regeneration. Sustainable, biologically active biomaterials capable of addressing both cutaneous and bone repair remain highly desirable. Here, we developed three-dimensional (3D) printed sodium alginate (SA) and alginate/hydroxyapatite (SA/HAP) hydrogels incorporating microalgae-derived extracellular vesicles (MdEVs) obtained from*Ettlia oleoabundans*. The constructs were characterized for their mechanical, structural, and biological properties, and evaluated in vitro using human dermal fibroblasts (hDFs) and mesenchymal stem cells (hMSCs). The printed hydrogels exhibited a well-defined architecture, mechanical stability, and high biocompatibility. Notably, the same bioactive agent, MdEVs, elicited distinct cell-type-specific regenerative programs depending on the material niche in which it was presented. MdEV-loaded SA scaffolds enhanced cell viability and activated AKT/mTOR signaling and extracellular matrix (ECM)-remodeling pathways in hDFs, supporting cutaneous repair. In contrast, MdEV-loaded SA/HAP scaffolds stimulated pro-angiogenic and osteoinductive gene expression in hMSCs, indicative of bone-regenerative potential. This differential bioactivity underscores the sophistication of the platform beyond simply promoting repair, demonstrating how material composition can direct context-dependent cellular responses by using a single, sustainable biological cue. Overall, this in vitro study demonstrates that MdEV-enriched alginate-based scaffolds can differentially guide fibroblast and stem cell responses relevant to skin and bone regeneration. These findings highlight the potential of algae-derived extracellular vesicles as versatile bioactive components in next-generation regenerative biomaterials for complex wounds involving multiple tissue types.

## Graphical abstract

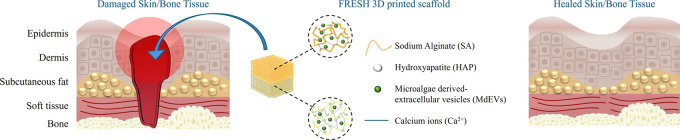

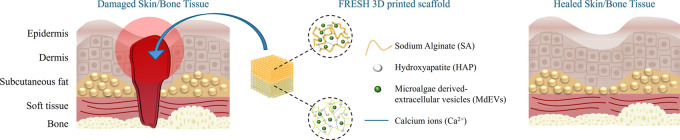

## 1 Introduction

Skin wounds typically undergo a timely and orderly reparative process based on four phases: hemostasis, inflammation, proliferation, and dermal remodeling, leading to anatomical and functional restoration, normally within a month. Chronic wounds fail to progress through these normal stages and persist for extended periods, often exceeding 3 months. Several intrinsic and extrinsic factors can contribute to the chronicity of wounds, including advanced age, hyperlipidemia, diabetes, infection, and peripheral vascular disease.^1^ All wounds initially exist as acute but may become chronic if the healing process is interrupted, particularly during the inflammatory phase, where a continuous state of inflammation triggers a cascade of tissue responses that prolong the nonhealing state.^2^ Some typical characteristics of chronic wounds are excessive production of pro-inflammatory cytokines, recurrent infections, biofilm formation, which increases bacterial resistance and consequently reduces the effectiveness of antibiotic treatments as well as the presence of senescent cells and necrotic tissues, which impair the response to reparative stimuli.^3,4^ Chronic ulcers, particularly in diabetic or elderly patients, are among the leading causes of long-term hospitalization and limb amputation, with substantial socioeconomic impact.^5^

One of the most severe complications of diabetic wounds is bone exposure, which can be caused either by the injury itself or by treatments aimed at promoting the healing process, such as debridement.^6^ Debridement is a commonly used technique for wound management, and it may extend to the subcutaneous tissue to remove necrotic tissue and bacterial biofilm.^7^ For wounds with bone exposure or large tissue loss, skin grafting has been proposed; however, direct bone coverage is challenging without the formation of granulation tissue over the structure, and additional limitations involve pain, itching, and excessive contraction during healing, which can result in scarring. Conventional nonsurgical wound treatments include various wound dressings, topical agents, scaffold- or hydrogel-based skin grafts, and skin substitutes.^8^ These approaches support wound care by maintaining appropriate moisture levels, controlling infection and inflammation, and regulating both re-epithelialization and tissue contraction.^9^ Despite their utility, conventional monofunctional dressings often fail in complex wounds with bone exposure, as they lack the necessary mechanical and biological cues to simultaneously drive osteogenesis and soft tissue repair. Similarly, while bone grafts address the skeletal deficit, they do not provide an adequate substrate for the overlying dermal regeneration, frequently leading to poor integration and high recurrence rates. There is, therefore, a critical need for multifunctional, bilayered platforms capable of providing site-specific modulation for both skin and bone compartments.^10^

Hydrogels are particularly attractive as wound-healing materials due to their high water content, tissue-like elasticity, and ability to deliver bioactive molecules in a controlled manner.^8,11,12^ Sodium alginate (SA), a marine-derived polysaccharide, is widely used for both soft and hard-tissue engineering. In this work, we used advanced three-dimensional (3D) printing based on the freeform reversible embedding of suspended hydrogels (FRESH) method to fabricate SA-based scaffolds with precise geometry and tunable porosity suitable for wound environments.^13^ This method involves the deposition and incorporation of SA and alginate/hydroxyapatite (SA/HAP) bioinks within a secondary hydrogel that serves as a temporary, thermoreversible, and biocompatible support. The support bath consists of gelatin microparticles that behave as Bingham plastic during the printing process, acting as a rigid body under low shear stress but flowing like a viscous fluid under higher shear stress. Additionally, the gelatin bath contains divalent calcium ions, which cross-link the alginate during the printing process. In this view, a biologically active hydrogel system was made of SA, enriched with extracellular vesicles (EVs), namely, exosomes, derived from a green microalga, introducing a novel bioactive component with broad regenerative potential. Recently, EVs have emerged as natural nanocarriers capable of mediating intercellular communication and promoting tissue repair. While mammalian EVs have shown promising results, their clinical translation remains limited by ethical and scalability issues. Microalgae-derived EVs (MdEVs) offer a sustainable and immunologically inert alternative. Their ability to stimulate fibroblast activity and influence osteogenic differentiation makes them particularly suited for dual soft- and hard-tissue repair.^14^ Recent studies have demonstrated that microalgal EVs exhibit high biocompatibility, anti-inflammatory, pro-angiogenic, and antioxidant properties, making them highly attractive for applications in wound healing.^15^ Their ability to modulate cytokine release, and enhance collagen production positions them as a valuable tool for cutaneous regeneration. Moreover, emerging evidence suggests their potential to interact with osteoblasts and preosteogenic cells, indicating a promising role in bone tissue repair and remodeling.^16^

The aim of this work was to develop and characterize 3D printed SA-based hydrogels incorporating MdEVs as bioactive agents and to evaluate their in vitro effects on human fibroblasts and mesenchymal stem cells as representative models of skin and bone healing. This study establishes a sustainable, animal-free proof of concept for dual tissue regeneration based on MdEVs-enriched biomaterials.

## 2 Materials and Methods

### 2.1 Materials

Gelatin (type A, bloom 300), calcium chloride (CaCl_2_), alginic acid sodium salt (SA, 15–25 cP, 1% in H_2_O), hydroxyapatite (HAP, nanopowder <200 nm particle size), phosphate-buffered saline (PBS), ethanol, glutaraldehyde, paraformaldehyde, dimethyl sulfoxide (DMSO), isopropanol, 3-(4,5-dimethylthiazol-2-yl)-2,5-diphenyltetrazolium bromide (MTT) reagent, and red fluorescent PKH26 labeling kit were from Sigma-Aldrich, Milan Italy. Phenol red-free Dulbecco’s modified Eagle’s medium high glucose (DMEM-HG), fetal bovine serum (FBS), penicillin–streptomycin (P/S) solution, Pierce bicinchoninic acid (BCA) assay kit were from Thermo Fisher Scientific, Milan Italy.

### 2.2 Preparation and Characterization of FRESH 3D Printing Support Bath

The gelatin microparticles, constituting the support bath, behave like Bingham plastic; flow like a viscous fluid under high shear stress, and act as a rigid body at low shear stress. This means that, when a needle-like nozzle moves through the bath, there is minimal mechanical resistance, but, at the same time, the extruded hydrogel, deposited within the bath, remains in place. As a result, soft materials are easily maintained in the intended 3D geometry. Additionally, the gelatin bath contains divalent calcium ions, which cross-link the alginate during the printing process. Moreover, their ability to encapsulate and gradually release therapeutic molecules, such as growth factors, nucleic acids, and nanoparticles, further enhances their regenerative potential. To create the gelatin slurry support bath, an adapted protocol from Hinton et al.^13^ was followed. Briefly, 200 mL of 4.5% w/v gelatin was dissolved in CaCl_2_ (0.1 M) and then gelled overnight at 4 °C. Next, 350 mL of CaCl_2_ at 4 °C was added to the cold gelatin, and the content was blended (at “pulse” speed) for a period of 120 s by using a consumer-grade blender (Waring Commercial). Then, the blended gelatin slurry was loaded into 50 mL conical tubes and centrifuged at 3500 rpm for 3 min, causing precipitation of slurry particles. The supernatant was removed and replaced with CaCl_2_ at 4 °C. The slurry was vortexed back into suspension and centrifuged again. This process was repeated until no bubbles were observed at the top of the supernatant, which indicated that most of the soluble gelatin was removed. After that, gelatin slurry was stored at 4 °C (Figure 1, step 1).

**Figure 1:**
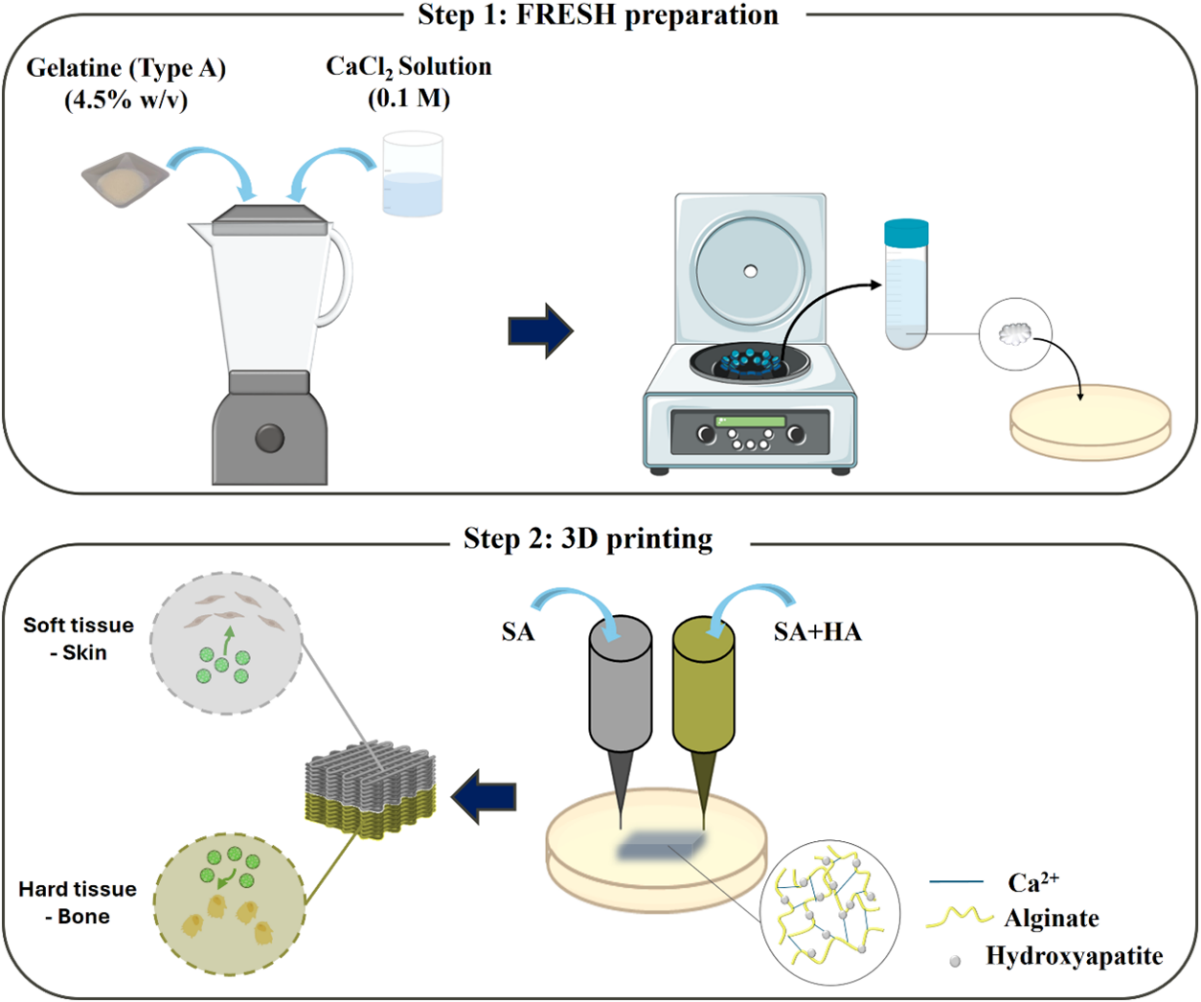
Representative scheme of scaffold biofabrication: (step 1) the gelatin-based FRESH preparation; (step 2) 3D printing of the dual-layer scaffolds based on alginate (SA) and alginate/hydroxyapatite (SA/HAP) bioinks. FRESH-printedSA-based scaffolds incorporating Microalgae-derived extracellular vesicles (MdEVs) provide a sustainable, dual-function platform for soft and hard-tissue regeneration.

### 2.3 Scaffolds 3D Printing Process

For FRESH printing, the slurry was poured into a Petri dish. Any excess fluid was removed from the gelatin slurry support bath using wipes, obtaining a slurry material that behaved like a Bingham plastic. The fabrication of neat and nanocomposite scaffolds was performed by using a 3D printing machine (ROKIT Dr. INVIVO 4D2, Rokit Healthcare, Seoul, Republic of Korea).

First, the SA concentration was optimized. To this aim, neat scaffolds were produced by using SA at different concentrations, 8, 12, and 16% w/v. Once the concentration of SA was selected, based on mechanical and morphological analyses, nanocomposite bioinks were produced. SA, at the selected concentration, was mixed with HAP at 15 and 30% compared to SA weight (w_SA_). The mixture was stirred vigorously for 48 h at room temperature (RT) until homogeneous pastes were achieved. The paste was loaded into a cartridge equipped with a 21 G needle size (internal diameter 0.51 mm) (Figure 1, step 2).

The final scaffold model was sliced using NewCreatorK 1.57.70 (Rokit Healthcare Inc., Seoul, Republic of Korea). Uninterrupted neat and nanocomposite strands were obtained under the conditions of a nozzle moving speed of 5 mm × s^–1^ at 25 °C. Neat and nanocomposite patches with predesigned morphology (square-shaped lattice) and dimensions (15 mm × 3 mm thickness) were 3D printed at RT, while the collecting plate was maintained at −4 °C to ensure optimal gelation and structural fidelity. The extrusion pressure was adjusted based on the inorganic content of the bioink to compensate for the increased viscosity: 336 kPa for the pure SA layer, 360 kPa for the SA/HAP 15% layer, and 393 kPa for the SA/HAP 30% layer. The porosity in the designed scaffolds was set at 70%. The orientation angle of the fibers was 0/90°, meanwhile, line patterns and 0.4 mm layer thickness were used. After 3D printing, the 3D composite scaffolds were washed in CaCl_2_ (0.1 M) at 80 °C, to remove the gelatin, and further cross-link the scaffold. Afterward, scaffolds were frozen at −80 °C and lyophilized (LaboGene’s CoolSafe 55-4, PRO, Bjarkesvej, Denmark). Crucially, MdEVs were loaded onto the printed scaffolds postfabrication and liophilization. This postprinting functionalization strategy was adopted to safeguard the MdEVs from extrusion-related shear stress and to enhance the clinical translatability of the platform by allowing for separate storage and improved shelf life of the components.

### 2.4 Scaffolds Evaluation

#### 2.4.1 Dynamic Mechanical Analysis

3D porous square scaffolds (length, 15 mm; thickness, 5 mm) were analyzed by dynamic mechanical analysis (DMA, TA-Q800, TA-Instrument, New Castle, DE, USA) in frequency sweep mode. The frequency discrete value was set at 1.0 Hz; meanwhile, an amplitude of 15 μm in compression, a preload of 0.01 N, and a force track of 125% were adopted. The tests were carried out in a closed chamber in a wet state at RT.

#### 2.4.2 Thermogravimetric Analysis

Thermogravimetric analysis (TGA) was performed to evaluate the thermal stability and the effective weight percentage of the HAP inside the nanocomposite scaffolds using a TA Instruments TGA model 2950. Dried specimens (4–7 mg) were heated under a nitrogen (N_2_) flow, from 20 to 900 °C at a heating rate of 10 °C × min^–1^.

#### 2.4.3 Morphological and Physicochemical Analyses

3D printed scaffolds were observed by scanning electron microscopy (SEM, FEI Quanta 200 FEG, Hillsboro, OR, USA). Scaffolds were washed with distilled water, frozen at −80 °C, and lyophilized. Subsequently, the lyophilized samples were coated with an ultrathin layer of Au/Pt by using an ion sputter and observed by SEM. SEM-energy-dispersive X-ray spectroscopy (EDS) mapping was also used to assess the dispersion of the HAP fillers in the polymer matrix. To further confirm the presence of HAP, attenuated total reflectance Fourier-transform infrared (ATR-FTIR) spectroscopy (Thermo Fisher Nicolet IS10, Waltham, MA, USA) was also employed. Neat and nanocomposite materials were scanned from 400 to 4000 cm^–1^ with a resolution of 4 cm^–1^. HAP was used as a control.

#### 2.4.4 Swelling and Stability Test

Dried scaffolds were weighed (*w*_0_) and left to swell in sterile DMEM-HG supplied with antibiotics (pH 7.4, T = 37 °C, V = 4 mL, up to 14 days) mimicking the physiological conditions. To assess their retention capacity, the swollen hydrogels were then taken out at fixed time points and quickly blotted on a filter paper to remove the superficial adsorbed solution, the weight was recorded (*w*_t_) and the samples were placed in medium again. The swelling ratio (*Q*) was calculated according to eq 1

1 $$Q=\left(\frac{w_{t}-w_{0}}{w_{0}}\right)$$

Stability was assessed by immersing the structures in sterile DMEM-HG for up to 21 days (pH 7.4, T = 37 °C, and V = 4 mL). At 7, 14, and 21 days, the structures were removed from the medium, washed in water three times, lyophilized, and reweighed. Stability was assessed using the following eq 2

2 $$\Delta P\;\left(\%\right)=\frac{{w_{0}-w}_{t}}{w_{0}}\times 100$$

where *w*_0_ denotes the weight of the sample before immersion in medium, *w*_t_ denotes the weight of the sample at time *t*, and Δ*P* (%) the percentage weight change.

### 2.5 Scaffolds Sterilization

To perform the different biological characterizations, the 3D printed scaffolds needed to be sterilized. Sterilization was achieved by washing the scaffolds with 75% ethanol and completely submerging them in the solution for 30 min. The scaffolds were then allowed to dry under sterile conditions in a laminar flow hood and further sterilized by exposure to a UV lamp for an additional 30 min. Scaffolds were then stored at 4 °C, under sterile conditions, in a Petri dish signed and sealed with Parafilm.

### 2.6 3D Printing as Proof of Concept of a Bilayer SA-SA/HAP Scaffold

The bilayer structure was achieved by sequential deposition of the respective bioinks within the gelatin slurry bath, starting with the SA/HAP layer to ensure a stable foundation, followed by the deposition of the SA layer. Printing parameters such as nozzle size, speed, extrusion pressure, and substrate temperature were maintained as previously optimized to preserve structural definition. Following printing, the scaffolds underwent a dual-phase postprocessing procedure involving thermal gelation of the support bath and ionic cross-linking, as described for single-layer scaffolds.

### 2.7 Extracellular Vesicles from Algae Cultures

A stock culture of the green microalgae *Ettlia oleoabundans* UTEX 1185 (syn. *Neochloris oleoabundans* UTEX 1185; www.utex.org) was cultivated in borosilicate glass flasks using BG-11 medium (for recipe: utex.org/products/bg-11-medium) until reaching the stationary phase of growth and a cell density of (20–22) × 10^6^ cells × mL^–1^. Cultures were maintained under white light-emitting diode (LED) light providing 60 μmol × m^–2^ s^–1^ of photosynthetic active radiation (PAR), with a 16:8 h light/dark photoperiod, and at a temperature of 24 ± 1 °C. Experimental cultures were then set up in triplicate by inoculating cells from the stock culture at a starting density of 1 × 10^6^ cells × mL^–1^, and maintained at the same conditions. After 28 days, an aliquot from each culture was collected and centrifuged at 300*g* for 10 min to separate the biomass from the supernatant (microalgae-conditioned medium; MCM). The MCM was then transferred to a clean tube for MdEVs isolation.

### 2.8 Isolation and Characterization

Isolation and characterization of MdEVs were carried out according to the protocols described by Trentini et al.^17^ The MCM underwent multiple centrifugation steps to progressively remove debris of increasing density. Initially, the samples were centrifuged at 650*g* for 10 min, followed by a second centrifugation at 3000*g* for 30 min, and a third at 10 000*g* for 1 h. The resulting supernatant was filtered through a 0.22 μm vacuum filtration unit (Sartorius, Göttingen, Niedersachsen, Germany) to further improve the sample purity. The filtrate was then subjected to an additional centrifugation step at 37 500 rpm for 1 h, using an Optima L-70 Ultracentrifuge equipped with a 70 Ti rotor (Beckman Coulter Inc., CA, USA). Tunable resistive pulse sensing (TRPS) was employed to assess the concentration and size of the MdEVs. The analysis was carried out using the qNANO Gold instrument (Izon Science Ltd., Christchurch, New Zealand) with NP150 nanopore stretched to 49 mm and measuring at two pressure levels (10 and 20 atm) with a particle rate above 200 particles min^–1^ and a total count exceeding 500. Calibration particles (CPC200, Izon Science Ltd.) were measured at both pressures and used to calibrate the sample data. The protein content of the MdEVs was determined using the BCA assay kit according to the manufacturer’s protocol. Absorbance at 570 nm was measured with a Victor 3 multilabel plate reader (PerkinElmer, Milan, Italy).

### 2.9 Morphology Evaluation

MdEVs were fixed in 1 mL of 2% glutaraldehyde in a phosphate buffer. The fixed particles were allowed to settle by gravity for 1 h on a clean coverslip at RT. For SEM analysis, samples were dehydrated through a graded ethanol series (50, 70, 80, 90, and 100%). The coverslip was then mounted on a suitable holder and coated with gold according to the standard protocols. Imaging was conducted under a high vacuum using a secondary electron detector with a Zeiss EVO 40 SEM (Zeiss, Oberkochen, Germany).

### 2.10 Fluorescent Labeling of Extracellular Vesicles and Confocal Imaging

The lipophilic fluorescent dye PKH26 was employed to label the MdEV membrane. Following a modified version of the supplier’s instructions, 0.8 μL of the PKH26 reagent was initially diluted in 200 μL of Diluent C and mixed with MdEVs previously suspended in PBS. The total volume was adjusted to 400 μL by the addition of further diluent. The staining reaction was carried out by incubating the mixture at RT for 5 min. Following this step, the vesicles were subjected to purification using centrifugal ultrafiltration devices with a 30 kDa cutoff (Amicon Ultra-0.5, Millipore, Burlington, MA, USA) to eliminate the unbound dye. Centrifugation was performed at 14 000*g* for 20 min.

Labeled vesicles as well as PBS (control) were subsequently incubated with human dermal fibroblasts (hDFs, ATCC, USA) and human mesenchymal stem cells (hMSCs, Lonza Bioscience, Switzerland). Then, cells were fixed in 4% paraformaldehyde and counterstained with Alexa Fluor 488-conjugated phalloidin to visualize the cytoskeletal actin structures. Fluorescent images were acquired using a Nikon ECLIPSE Ti confocal laser scanning microscope equipped with a DS-Qi2 digital camera. Observations were conducted using 60× oil-immersion objectives.

### 2.11 Biocompatibility Assay

The biocompatibility of SA or SA/HAP scaffolds loaded with or without MdEVs was investigated by an in vitro cytotoxicity assay using the L929 murine fibroblast cell line (Interlab Cell Line Collection, Genova, Italy). Cells were seeded in 24-well culture plates at a density of 4 × 10^4^ per well and maintained for 24 h in DMEM-HG complete culture medium supplemented with 10% FBS and 1% P/S. After incubation with the scaffolds, the culture medium was aspirated and replaced with 1 mL of MTT reagent (0.5 mg × mL^–1^ in PBS). Samples were then incubated at 37 °C for 3 h to allow for formazan crystal formation, indicative of metabolically active cells. At the end of the incubation period, the MTT solution was carefully removed. To solubilize the formazan, 0.5 mL of a 10% DMSO/isopropanol solution was added to each well, and plates were incubated for 30 min at 37 °C. Subsequently, 200 μL aliquots from each well were transferred to a 96-well microplate, and absorbance was measured at 570 nm using a multimode plate reader (Victor 3, PerkinElmer, Milan, Italy). The resulting optical density (OD) values, obtained in duplicate, were used to determine relative cell viability across the experimental conditions.

### 2.12 Gene Expression Profiling via Real-Time Polymerase Chain Reaction (PCR)

hDFs and hMSCs were cultured in DMEM-HG with 10% FBS, 1% P/S at 37 °C in 5% CO_2_ and a humidified atmosphere. For gene expression analysis, 2 × 10^4^ hDFs were seeded in contact with the SA scaffolds for 7 days, whereas hMSCs were cultured with the SA/HPA scaffolds for 14 days. Before cell culturing, scaffolds were soaked with MdEVs or PBS (control condition) for 20 min, allowing the complete rehydration of the biomaterial.

Total RNA was extracted and purified by utilizing the RNeasy Mini Kit (Qiagen, Hilden, Germany), which included a DNA removal step using RNase-Free DNase, applied directly during column purification. All procedures were conducted following the manufacturer’s protocol. RNA concentration and purity were determined using a NanoDrop 2000 spectrophotometer (Thermo Fisher Scientific, Waltham, MA, USA). For reverse transcription, 500 ng of purified RNA from each sample were used to synthesize cDNA using the RT2 First Strand Kit (Qiagen). Reactions were carried out in a SimpliAmp Thermal Cycler (Applied Biosystems). Synthesized cDNA was subsequently preserved at −20 °C until downstream processing. Two pathway-focused PCR array panels (Qiagen), targeting genes involved in human wound repair and stem cell function, were selected for gene expression analysis. The cDNA samples were combined with SYBR Green-based RT2 qPCR Mastermix and dispensed into a 96-well plate preloaded with primer sets specific to each array. Quantitative PCR was performed using the StepOnePlus instrument (Applied Biosystems, Foster City, CA, USA), running an amplification protocol comprising an initial activation at 95 °C for 10 min, followed by 40 thermal cycles of 95 °C for 15 s and 60 °C for 60 s. Melt curve analysis was included at the end of the run, with the following profile: 95 °C for 60 s, 65 °C for 2 min, then continuous acquisition from 65 to 95 °C at an increment of 2 °C × min^–1^.

Gene expression levels were quantified by the 2^–ΔΔCT^ method. Ct values for target genes were normalized to the average expression of five internal reference genes: ACTB, B2M, GAPDH, HPRT1, and RPLP0. Fold change values were computed by comparing the expression levels in experimental groups to those in control groups. All gene quantification and normalization were executed using the GeneGlobe Data Analysis platform (Qiagen). Statistical interpretation was based on unpaired Student’s *t* tests applied to ΔCT values between sample groups. A *p*-value below 0.05 was considered statistically significant.

### 2.13 Enzyme-Linked Immunosorbent Assay (ELISA) Test

Samples were lysed to collect protein, centrifuged to remove cellular debris, and then stored as aliquots until further analysis. The levels of interleukin-10 (IL-10), IL-6, vascular endothelial growth factor (VEGF), and collagen I were determined using commercially available human ELISA kits (Invitrogen, Thermo Fisher Scientific, Frederick, MD) in accordance with the manufacturer’s instructions. Absorbance was read at 450 nm by using a Multiskan FC microplate reader (Thermo Fisher Scientific). Results are represented as a percentage compared to the control condition.

### 2.14 Statistical Analysis

Statistical analyses as *t* tests were performed on data through GraphPad Prism 8 (GraphPad Software Inc., USA), allowing determination of the statistical significance of the data in terms of *p*-value. For gene expression data analysis, Q-Rex Software (Qiagen) was used to define the fluorescence threshold value and collect Ct values. The ΔΔCt method was applied to the Ct values, and relative gene expression was defined as Fold Change (FC = 2^–ΔΔCt^), which was obtained for each sample by normalizing to a housekeeping gene and comparing the normalized sample data to the control.^18^

## 3 Results and Discussion

### 3.1 Optimization of SA Concentration

Mechanical properties represent one of the most important parameters to consider in the design of scaffolds for bone and skin wound healing. Indeed, the structures for skin–bone regeneration must provide a bilayered structure, with a soft, elastic upper layer for skin and a stiffer lower layer for bone. The mechanical gradient between layers ensures both flexibility for soft tissue and strength for bone support. Specifically, the structures must possess a mechanical behavior matching that of skin, with Young’s moduli ranging from 10 to 50 kPa, meanwhile, high tensile and compressive strength ensuring stability during both in vitro and in vivo cell growth processes are required to support bone tissue regeneration.^19^ Different studies demonstrated that elastic moduli (>100 kPa) are useful to favorably stimulate fibroblasts growth.^20,21^ This elastic region may thus serve as a mechanical transition zone, supporting soft tissue regeneration while transferring appropriate mechanical cues to the underlying, stiffer bone-regenerative phase. For effective osteogenic stimulation, however, the bone layer typically requires a modulus several orders of magnitude higher. Results proved that it was possible to successfully print SA structures at concentrations ranging from 8 to 16%. DMA results performed on SA scaffolds at different concentrations (8, 12, and 16% w/v) showed values of storage modulus (*E*′), which generally depended on SA amount. Particularly, *E*′ spanned from 19.9 kPa (8% w/v) to 62.4 kPa (16% w/v) at 1 Hz (Figure 2A). Those results are in line with the ones obtained from Majhi et al.^20^

**Figure 2:**
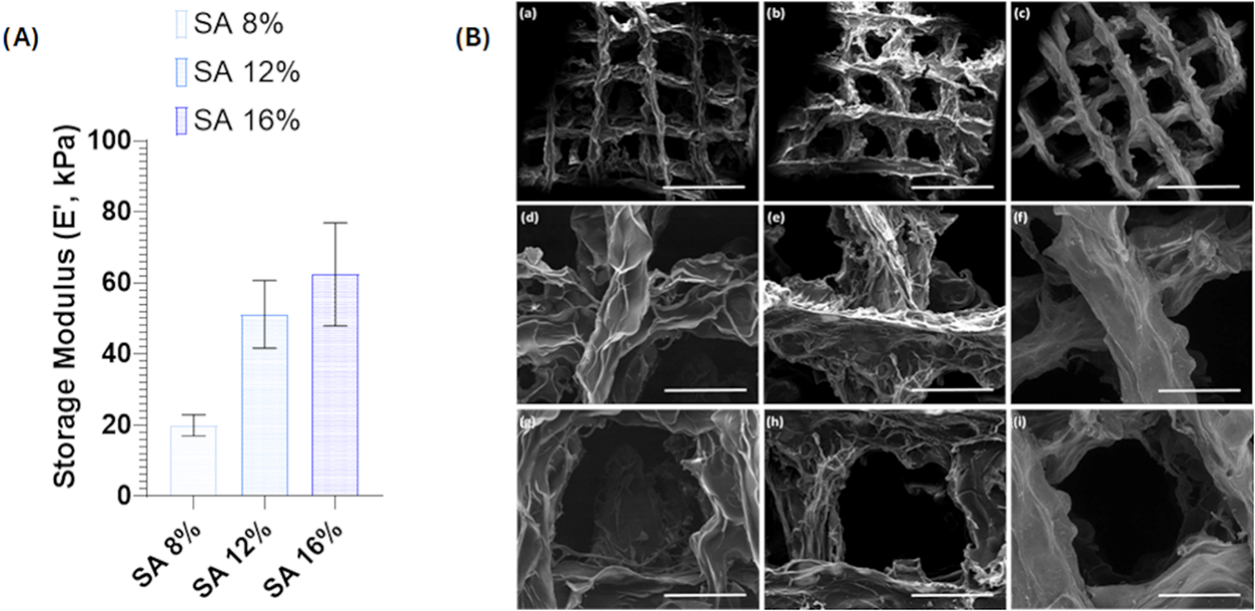
Optimization of the mechanical and morphological properties of neat SA scaffolds: (A) storage modulus of SA (8, 12, and 16% w/v); (B) scanning electron microscopies of (a, d, g) SA 8% w/v, (b, e, h) SA 12% w/v, and (c, f, i) SA 16% w/v. Scale bars: (a–c) 2 mm; (d–i) 500 μm.

SEM analysis (Figure 2B) of the SA 8, 12, and 16% scaffolds revealed the overall morphology (Figure 2B­(a–c)), providing detailed insights into the interwoven fiber network (criss-cross fibers) (Figure 2B­(d–f)) and the distribution of the pores (Figure 2B­(g–i)). The SEM micrographs revealed well-defined, interconnected porous networks characteristic of 3D printed SA scaffolds. Across all samples, the grid-like macrostructure was preserved, indicating good shape fidelity after printing and cross-linking. The filaments appeared continuous, and the junctions between printed layers were well-defined, suggesting stable layer adhesion during the fabrication process. At higher magnifications, the strut surfaces exhibited a wrinkled and sheet-like morphology, typical of dried hydrogel materials. The internal pores were open and interconnected, forming a highly porous architecture that would be favorable for cell infiltration and nutrient diffusion. Although all samples displayed similar overall structural features, slight variations in filament thickness and pore uniformity can be observed, which may be influenced by differences in the SA concentration and the resulting viscosity during printing. Overall, the scaffolds demonstrated a balance between structural integrity and porosity, with a well-preserved 3D architecture and microscale roughness that could support cell attachment. Based on these results, the SA 16% formulation was selected for further development, because it showed the highest mechanical performance for the skin layer. This composition was subsequently functionalized with HAP to enhance its bioactivity and compatibility with the underlying bone-regenerative environment. From now on and throughout the manuscript, SA 16% scaffolds will be denoted with SA.

### 3.2 Characterization of Nanocomposite Scaffolds

DMA analysis was also performed on nanocomposite scaffolds (Figure 3A). Results highlighted the effect of HAP presence on the storage moduli (*E*′). Indeed, HAP acted as reinforcement of the polymer matrix, showing *E*′ values spanning from 62.4 kPa (SA) to 82.2 kPa (SA/HAP 30%). There were no statistical differences between SA/HAP 30% and SA/HAP 15%. It is worth noting that the storage modulus of the developed hydrogels (∼80 kPa) is substantially lower than that of native cortical bone, which lies in the GPa range. However, the aim of the present work is not the regeneration of large, load-bearing bone defects, where mechanical reinforcement and structural support are essential. Instead, the developed platform was designed for the treatment of small bone lesions and exposed bone areas associated with chronic skin wounds (i.e., diabetic ulcers), where the primary function of the material is biological instruction rather than mechanical load bearing. In this context, the hydrogel does not act as a structural substitute for bone but rather as a temporary, bioactive reservoir or filler that conforms to defect geometry, maintains a moist environment, and locally delivers regenerative cues to stimulate endogenous repair processes. This strategy is well aligned with a large body of literature demonstrating the successful use of soft hydrogels for bone-related applications, particularly when the goal is to promote osteoinduction, angiogenesis, and cell recruitment rather than to provide mechanical stability.^22,23^ In such approaches, mechanical fixation or the surrounding native tissue bears the load, while the hydrogel facilitates biological regeneration. Moreover, the inclusion of HAP within the SA matrix may provide biochemical and osteoinductive cues that are known to support osteogenic differentiation and mineralization independent of bulk mechanical stiffness. The relatively low modulus may also be advantageous in promoting cell infiltration, nutrient diffusion, and vascularization, which are critical for bone healing in nonload-bearing or microdefect scenarios. Therefore, while the mechanical properties of the hydrogels are not intended to match those of native bone, they are purposefully tailored to their intended clinical application, namely, the treatment of complex skin wounds with limited bone involvement, where biological activity and controlled release of bioactive components are the dominant design criteria.

**Figure 3:**
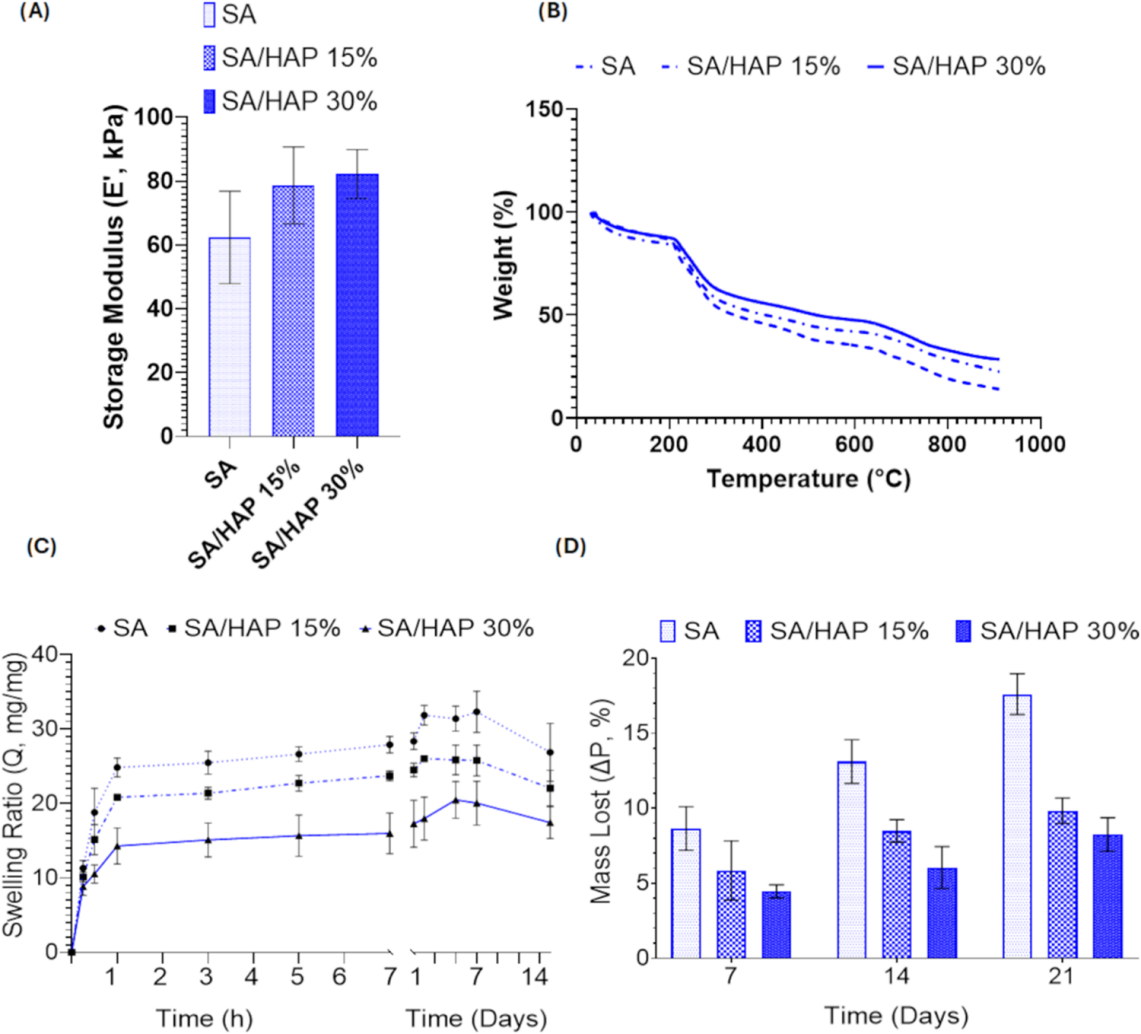
Characterization of the nanocomposite scaffolds. (A) Storage modulus of SA, SA/HAP 15% and SA/HAP 30% with SA set at 16% w/v. Results are expressed as mean value ± standard deviation. (B) Results from thermogravimetric analysis (TGA), (C) swelling ratio (*Q*) and (D) stability tests, in terms of mass lost, for neat and nanocomposite scaffolds.

The presence of HAP was also confirmed by TGA (Figure 3B). TGA of nanocomposite scaffolds was carried out at temperatures ranging from 20 to 900 °C under N_2_. Thermal decomposition of the SA-based scaffolds took place at temperatures of approximately 200 °C. The residual mass of 13.9 wt %, found at 900 °C for SA, might be related to the production of coke residue during thermal decomposition. As the ceramic phase is quite stable over 900 °C, the residual masses for SA/HAP 15% and SA/HAP 30% were 22.5 and 28.5 wt %, respectively, and they can be used to determine the amount of HAP present in the scaffolds.

Swelling and stability behaviors are important properties of scaffolds for wound healing applications (Figure 3C,D). Our scaffolds proved to be highly hydrophilic and able to rehydrate under physiological conditions (Figure 3C). However, by increasing the amount of HAP within the scaffold, the swelling ratio decreased. Indeed, scaffolds reached after 1 h a *Q* value of 24.8, 20.8, and 14.3 for SA, SA/HAP 15% and SA/HAP 30%, respectively. Those results suggest that the presence of HAP seems to prevent the complete hydration of the scaffold, contracting and restricting the polymer chain mobility. *Q* value remained constant until 14 days in agreement with the previous result reported by D’Amora et al.^24^

In terms of stability, the scaffolds maintained their lattice-like structure for up to 21 days (Figure 3D). Nanocomposite scaffolds proved to be more stable with a degradation of 8% (SA/HAP 30%), compared to neat SA, which showed a degradation of 18%.

### 3.3 SEM and EDS of Nanocomposite Scaffolds

Nanocomposite scaffolds highlighted the presence of HAP inside the SA matrix as evidenced by SEM and SEM-EDS analyses (Figure 4A–D). HAP appeared homogeneously distributed, partially exposed to the surface. This morphological aspect is of paramount importance since it may also affect cell response.

**Figure 4:**
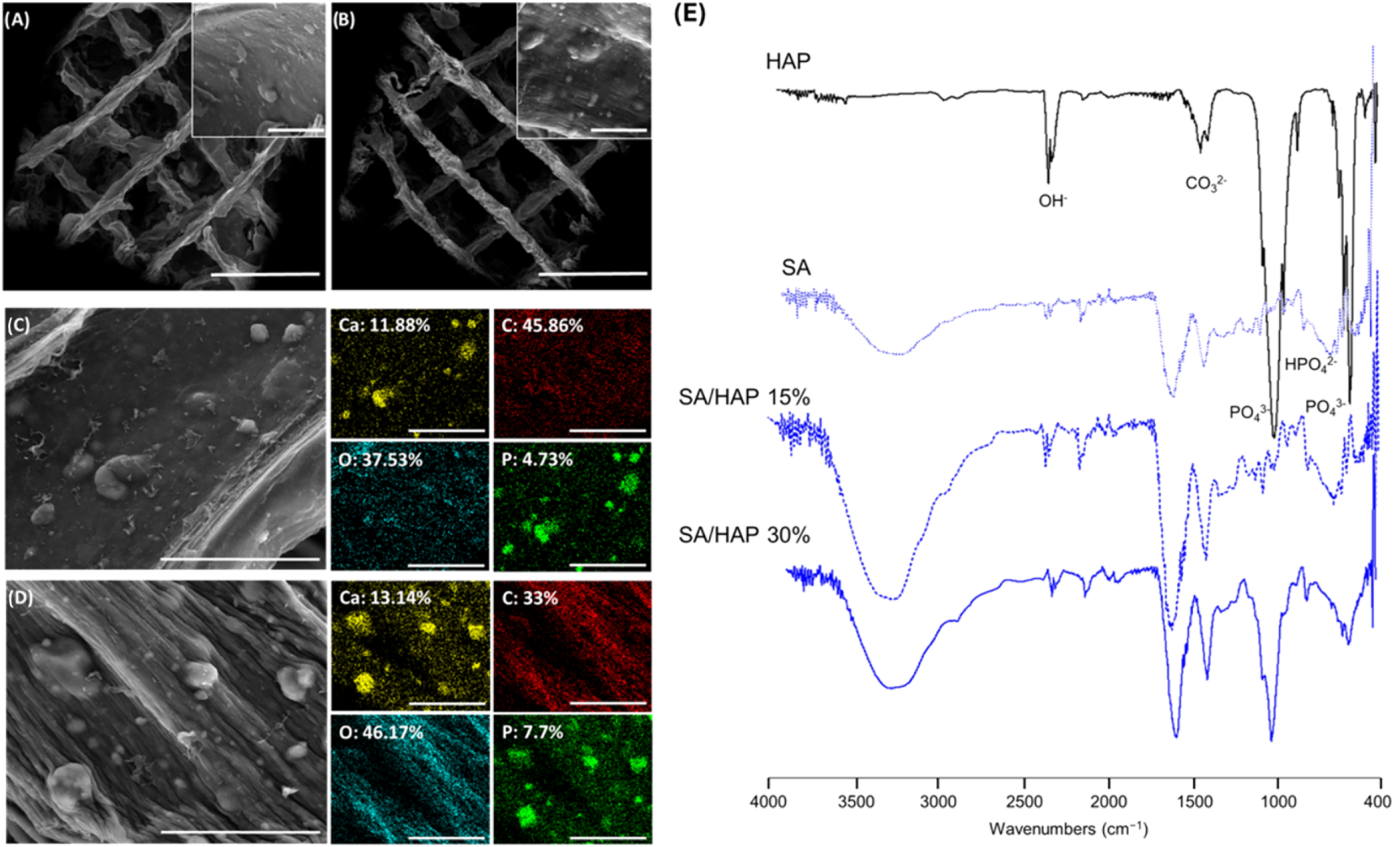
SEM, EDS, and ATR-FTIR spectra of nanocomposite scaffolds. SEM images of nanocomposite scaffolds: (A) SA/HAP 15%; (B) SA/HAP 30%. Scale bars: 2 mm. Insets, scale bars: 50 μm. SEM-EDS analysis of 3D printed scaffolds: (C) SA/HAP 15%; and (D) SA/HAP 30%. Scale bars: 90 μm. Ca stands for calcium, C for carbonium, O for oxygen, and P for phosphorus. (E) ATR-FTIR spectra of HAP, SA, SA/HAP 15%, and SA/HAP 30% between 4000 and 400 cm^–1^.

ATR-FTIR spectroscopy was employed to determine the existing functional groups of commercial HAP. The chemical groups in the FTIR spectrum of HAP are PO_4_^3–^, OH, and CO_3_^2–^ characteristic of HAP (Figure 4E). The strong complex broad bands at 1085 and 1017 cm^–1^ represent the stretching mode of the P–O vibration of the PO_4_ group in the HAP structure. The bands at 629 and 560 cm^–1^ in Figure 4D are the bending modes of PO_4_.^25,26^

The infrared spectrum of the SA showed a broad absorption band between 3500 and 3100 cm^–1^, referring to the stretching of the OH group. Specifically, the peaks at 3260, 1026, and 2926 cm^–1^ were assigned to stretching vibrations of OH, COC, and CH, respectively, confirming the typical polysaccharide structure. The strong peak at 1600 cm^–1^ and a somewhat weaker peak at 1415 cm^–1^ were attributed to the asymmetric and symmetric stretching vibration of the carboxylate group, respectively.^27^

The FTIR spectra of the SA/HAP composites showed characteristic absorption bands of both components. In addition to the typical alginate bands (O–H, C–H, and COO^–^ vibrations), new bands appear at ∼557 cm^–1^, corresponding to the bending vibrations of phosphate groups (PO_4_^3–^) in HAP. The broad band near 1031 cm^–1^, associated with PO_4_^3–^ stretching, is also intensified compared to neat SA. These features confirm the successful incorporation of HAP into the SA matrix without significant alteration of the alginate’s chemical backbone. Even if the ATR-FTIR is widely known and is not a quantitative analysis, those bands appear more intense in SA/HAP 30%.

### 3.4 3D Printing as Proof of Concept of a Bilayer SA-SA/HAP Scaffolds

As a final proof of concept, a bilayer scaffold composed of a pure SA upper layer and a SA/HAP nanocomposite bottom layer was fabricated using the FRESH 3D printing approach. This configuration was designed to simultaneously meet the mechanical and biological requirements for chronic wound treatment, providing structural integrity through the SA/HAP component and a highly hydrated, cell-friendly surface via the SA layer. SEM analysis confirmed the architectural fidelity and distinct stratification of the two layers, with the bottom SA/HAP layer exhibiting a denser microstructure due to the ceramic filler, while the upper SA layer retained a more porous and interconnected morphology (Figure 5A,B).

**Figure 5:**
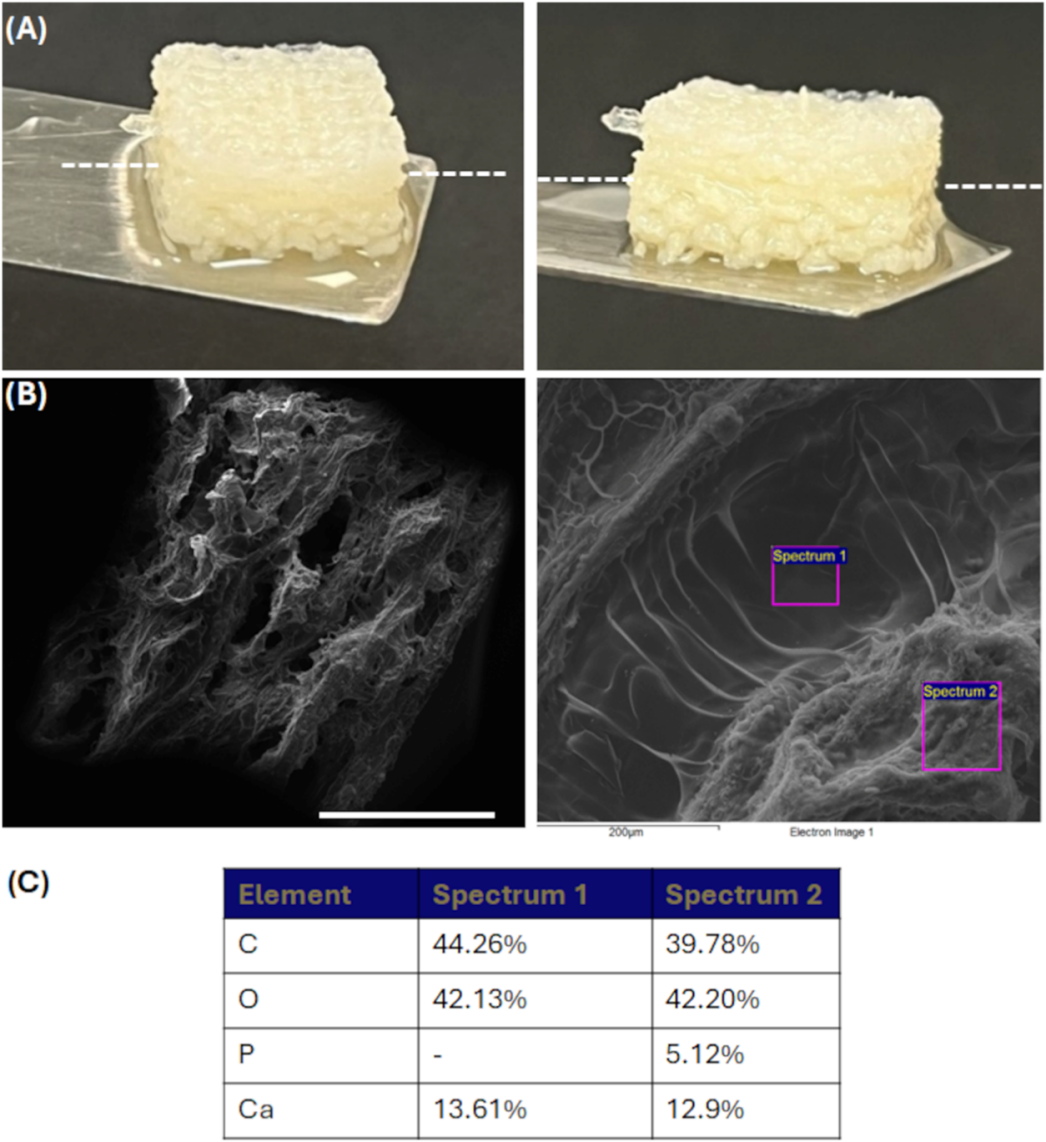
Bilayer SA-SA/HAP scaffold as a proof of concept of the 3D bioprinting technology. (A) Digital photograph highlighting the two different areas. (B) SEM image of the cross-section. Scale bar: 2 mm (left), 200 μm (right). (C) EDS analysis. Ca stands for calcium, C for carbonium, O for oxygen, and P for phosphorus.

This design demonstrated not only the feasibility of fabricating multimaterial scaffolds with tailored properties but also highlighted the potential for future developments in spatially controlled bioactive agent delivery. Furthermore, SEM performed on the cross-section at the interface between SA and SA/HAP areas confirmed that there was no delamination between the two compartments. Meanwhile, the only presence of P ions in the SA/HAP zone, detected by SEM-EDS analysis (Figure 5C), is indicative that the two zones were separated but closely interconnected with each other.

### 3.5 Characterization of MdEVs

The physical characterization of MdEVs was performed by TRPS, a technique capable of accurately measuring nanoparticle size, distribution, and concentration (particles × mL^–1^). The analysis revealed a population of vesicles with diameters ranging from 60 to 120 nm, in agreement with the expected size profile of small extracellular vesicles, including exosomes. The average particle diameter was calculated to be 86 nm, suggesting a uniform and stable vesicle preparation. SEM analysis revealed that MdEVs exhibited a generally spherical morphology with smooth surfaces and relatively uniform size distribution. The vesicles appeared well-dispersed on the coverslip with minimal aggregation. No significant structural abnormalities or debris were observed, indicating good preservation of vesicle integrity during sample preparation (Figure 6A,B).

**Figure 6:**
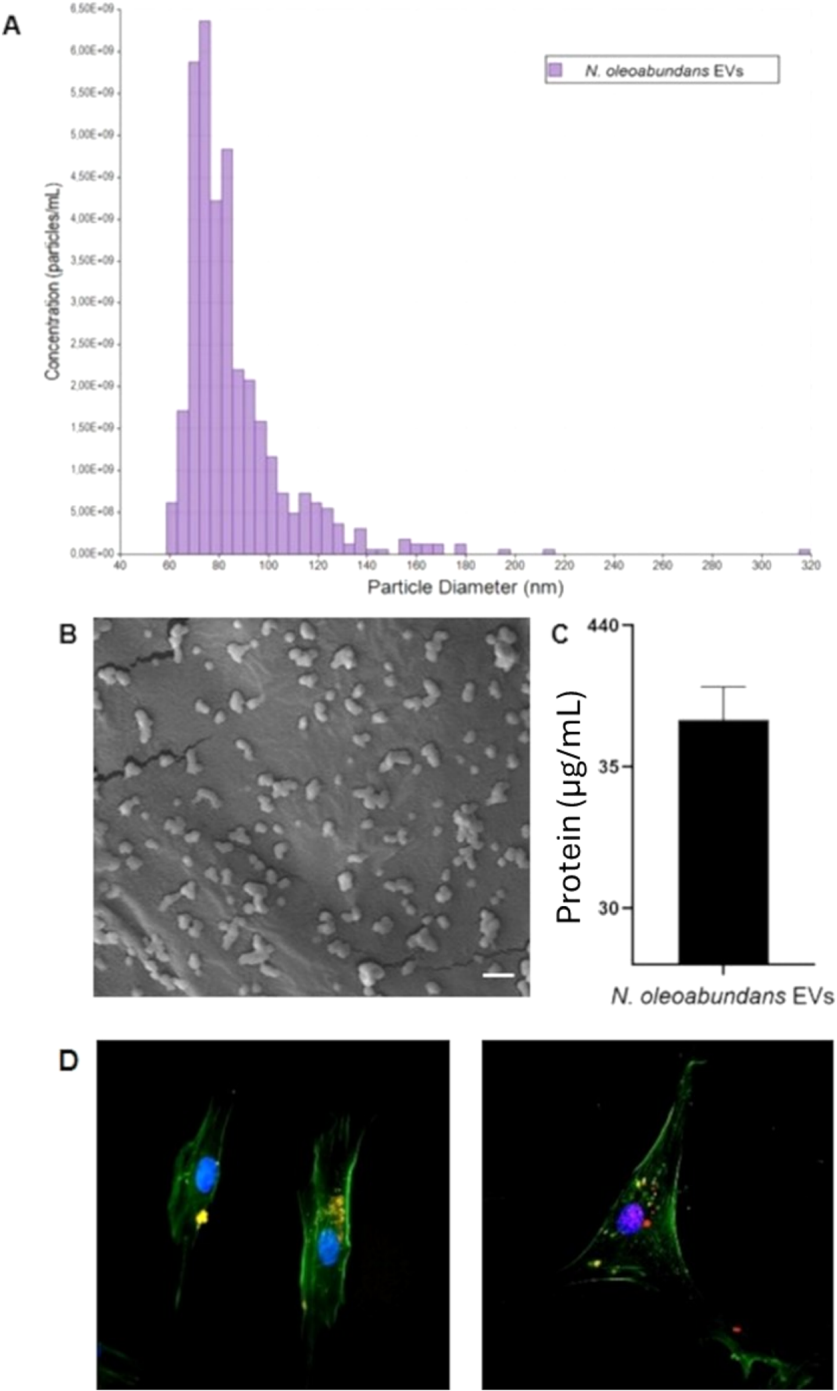
Characterization of microalgae-derived extracellular vesicles (MdEVs): (A) particle diameter distribution and concentration by tunable resistive pulse sensing (TRPS); (B) scanning electron microscopy. Scale bar: 1 μm; and (C) protein content quantified by BCA assay. (D) MdEVs uptake in human fibroblast (left panel) and mesenchymal stem cells (right panel): EV red; nuclei blue, cytoskeleton green.

Protein content was quantified using a standard BCA assay, yielding a concentration of 490 ng × μL^–1^ (Figure 6C). This value reflects the protein load typically associated with vesicle-enclosed cargo including surface and luminal proteins of biological relevance. The consistency in vesicle size and protein concentration indicates the successful isolation of a structurally intact EV population appropriate for functional assays. Fluorescence microscopy confirmed the successful uptake of PKH26-labeled MdEVs by human cells (Figure 6D). Following incubation, red fluorescence was clearly detected within the cytoplasm, indicating the internalization of the labeled EVs. The signal appeared to be punctual and was distributed throughout the perinuclear region, suggesting vesicle trafficking through the endocytic pathway.

### 3.6 SA-Based Scaffolds Loaded with MdEVs

The biocompatibility of the scaffolds was rigorously evaluated by using the MTT assay, a reliable and widely used colorimetric method that measures mitochondrial enzymatic activity as an indirect marker of cell viability and metabolic competence. This assay was selected for its sensitivity in detecting early cytotoxic effects as well as subtler differences in cell metabolic behavior in response to various biomaterial compositions.^28^ Precisely, the experimental setup included SA scaffolds and SA/HAP scaffolds with or without MdEV loading to evaluate the general cytocompatibility of the materials and the influence of EVs or mineral loading on cellular response. Cells were seeded in direct contact with scaffolds, and the MTT assay was performed at three time points (1-, 3-, and 7-days postexposure) to assess both the acute and progressive cellular response over time (Figure 7).

**Figure 7:**
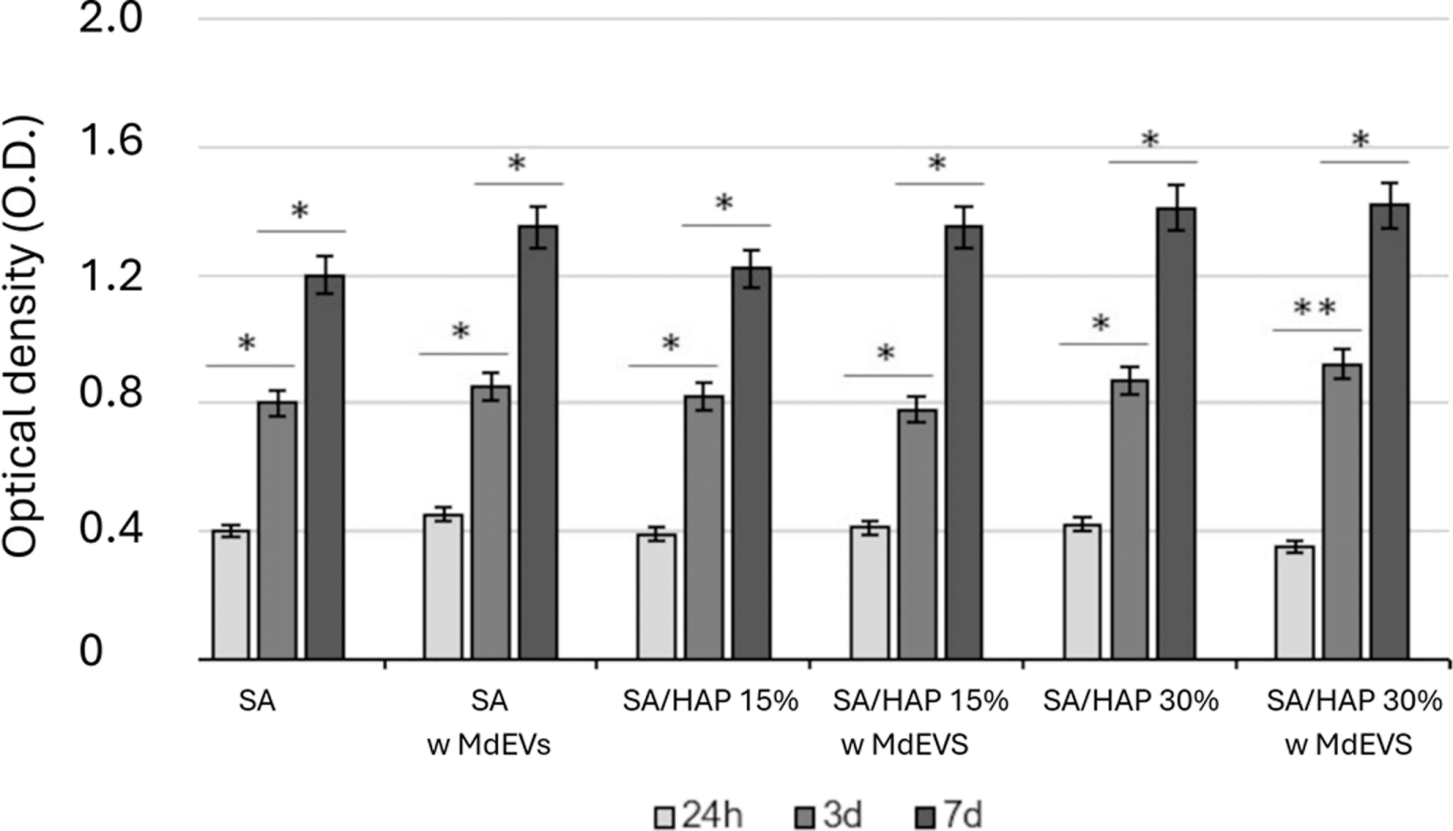
Biocompatibility assay. The graph reports the OD 570 nm derived from cells seeded on SA scaffolds, SA scaffolds loaded with microalgae-derived extracellular vesicles (MdEVs), SA/HAP 15% scaffolds, SA/HAP 15% scaffolds loaded with MdEVs, SA/HAP 30% scaffolds, and SA/HAP 30% scaffolds loaded with MdEVs. MTT assay was performed at 24 h, 3, and 7 days from the seeding. All data were recorded in triplicate, and results are expressed as mean ± standard deviation. *p-values <0.05; **p-value <0.01.

Across all groups, the presence of MdEVs was shown to be noncytotoxic, and in some conditions, particularly at day 3 and day 7, it was associated with a modest but consistent increase in metabolic activity. This suggests that the bioactive cargo of the vesicles supports a favorable microenvironment for cell survival and proliferation. More intriguingly, in the composite constructs containing HAP, especially SA/HAP 30% scaffolds, a marked enhancement in cell viability was observed, which became progressively more pronounced over time. This observation opens several possible interpretations. First, the increased roughness and porosity introduced by HAP particles may improve the initial cell attachment and nutrient exchange. Second, and more notably, HAP may function as a functional depot, promoting the adsorption, protection, and gradual release of MdEVs within the local microenvironment. This “reservoir effect” may allow for the sustained delivery of vesicular bioactive compounds, thereby prolonging their cellular influence and enhancing biological efficacy. This hypothesis is consistent with trends observed in controlled-release systems using mineral substrates as carriers for biomolecules. These results strongly support the biocompatibility and functional integration of MdEVs within both the SA and SA/HAP scaffolds. They also point to a synergistic interaction between MdEVs and HAP, with the mineral phase not only contributing to mechanical reinforcement and osteoconductivity but also potentially amplifying the vesicle-mediated biological effects.

### 3.7 Regenerative Potential of SA and SA/HAP Scaffolds

The SA and SA/HAP scaffolds have been manufactured for cutaneous and bone tissue regeneration purposes. To simulate cutaneous and bone tissue regeneration in vitro and determine the biological relevance of both substitutes, hDFs and hMSCs were seeded onto SA and SA/HAP scaffolds, respectively. In fact, hDFs have a central role in skin repair, extracellular matrix (ECM) deposition, and inflammatory modulation, whereas hMSCs are a well-established model for bone regeneration studies.^29–32^ The primary objective of this part of the study was to investigate the role of MdEVs incorporated into SA or SA/HAP scaffolds on the gene expression profile of cells seeded in each scaffold. In particular, the effect of EVs was screened to be consistent with the specific regenerative applications for which the scaffolds were designed. Gene expression analysis focused on the overall modulation of gene sets associated with tissue repair, cellular remodeling, and lineage-specific differentiation, without targeting a specific pathway. The approach was designed to detect early transcriptional changes that could reflect the initiation of pro-regenerative signaling cascades, which are critical for scaffold integration and functionality.

#### 3.7.1 Gene Expression on MdEV-SA Scaffolds

hDFs play a pivotal role in the complex cascade of events involved in skin wound healing. As the primary mesenchymal cells in the dermis, fibroblasts are responsible not only for producing ECM components (e.g., collagen and fibronectin) but also for coordinating the repair process via the secretion of cytokines and growth factors, as well as via direct responses to metabolic and environmental cues. A key regulatory axis that controls fibroblast behavior during tissue regeneration is the AMP-activated protein kinase (AMPK)-AKT signaling pathway. This pathway senses and responds to changes in cellular energy status and growth factor stimulation. Under stress conditions such as tissue injury, AMPK is activated to preserve energy homeostasis, while AKT modulates survival, migration, and anabolic activities. Together, these pathways ensure fibroblast proliferation, motility, and functional adaptation during wound closure and dermal remodeling.^33^

hDF in MdEV-SA scaffolds (Figure 8A) showed upregulation of AKT2 and MTOR genes that support enhanced cell survival, migration, and protein biosynthesis, which is essential for matrix deposition and tissue repair. Simultaneously, the increase in ADIPOR2 and PNPLA2 gene expression reflect a shift toward enhanced lipid metabolism, likely to meet the elevated energetic demands of active fibroblasts during repair. On the other hand, the downregulation of TP53 may relieve cells from apoptotic restraint, allowing greater proliferation under controlled conditions, while lower INSR expression could represent an adaptive shift toward insulin-independent metabolism, possibly driven by AMPK activity. These results indicate that fibroblasts orchestrate a highly dynamic transcriptional program that balances anabolic and catabolic demands to optimize their function during dermal wound healing. These findings could inform therapeutic strategies aimed at modulating fibroblast metabolism and signaling to enhance tissue regeneration, especially in chronic or nonhealing wounds.

**Figure 8:**
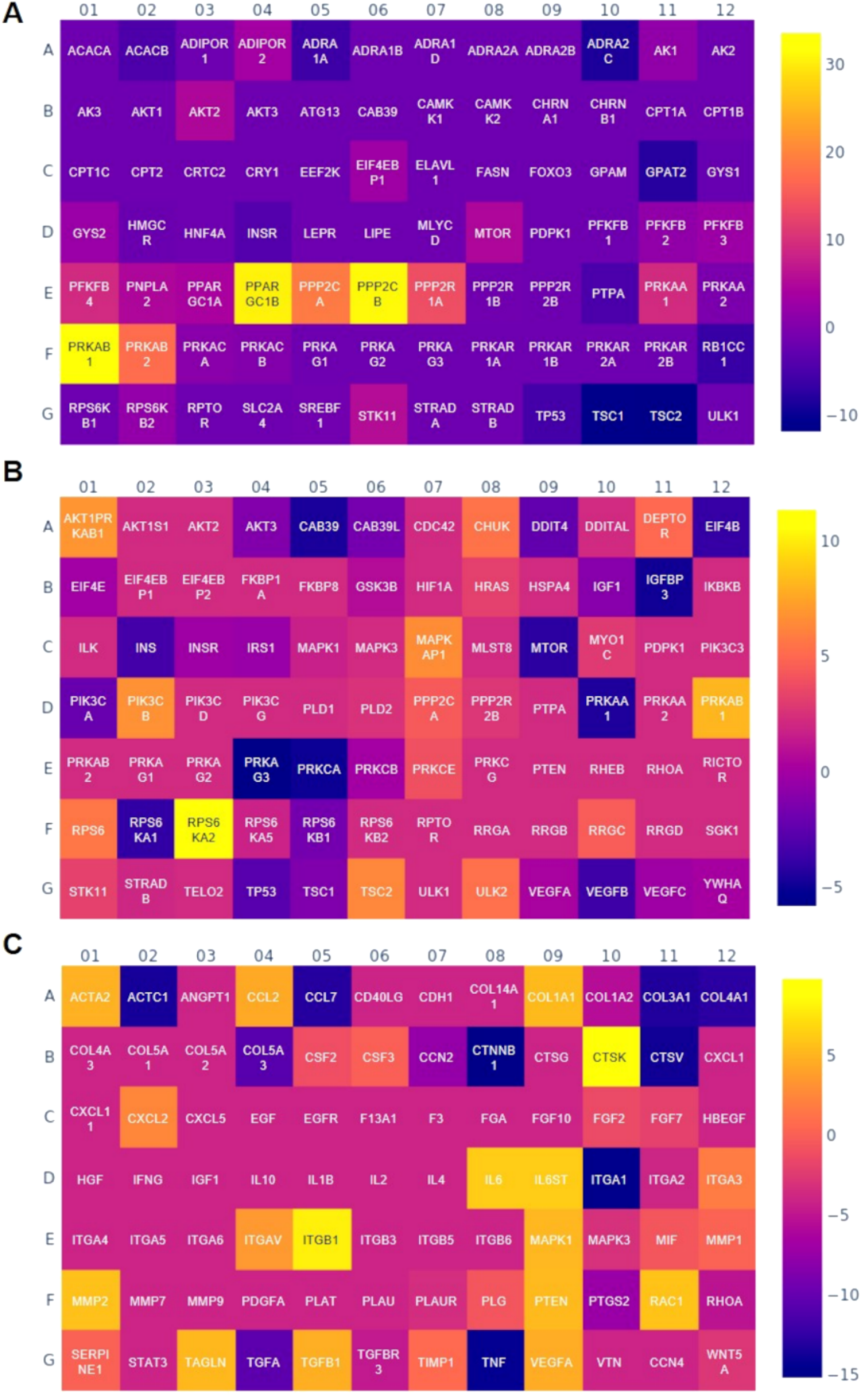
Gene expression profile of human fibroblasts on SA scaffolds loaded with microalgae-EVs compared with SA scaffolds. (A) AMPK-AKT signaling; (B) mTOR signaling; and (C) wound healing signaling. Heat map Log(2) fold change.

The mTOR signaling pathway is a central regulator of cellular metabolism, growth, and survival, playing a key role in tissue regeneration, including cutaneous wound healing. In dermal fibroblasts, mTOR acts as a metabolic and proliferative hub, integrating extracellular cues such as nutrients, growth factors, and cellular stress with intracellular energy status to drive appropriate regenerative responses. During wound healing, fibroblasts require tightly regulated mTOR activity to promote cell cycle progression, protein synthesis, and matrix remodeling, while also modulating responses to inflammation and hypoxia.^34^

hDF on MdEV-SA scaffolds demonstrated a distinct transcriptional response, consistent with an active mTOR-related program (Figure 8B). The transcriptional profile suggests a state of dynamic mTOR activation in fibroblasts. The concurrent upregulation of key effectors (AKT, RPS6, PIK3, MAPK) and modulators of energy balance (STK11, PRKAB, DEPTOR) points to an environment of controlled anabolic activity, balancing growth promotion with stress-responsive metabolic regulation. Interestingly, while mTORC1 outputs appear enhanced (e.g., protein synthesis and RPS6 activation), the downregulation of core mTOR transcripts (MTOR and CAB39) suggests a feedback-controlled system, possibly to prevent overactivation and maintain fibroblast homeostasis. Reduced expression of angiogenesis- and cell cycle-regulatory genes (VEGF, IGFBP3, and MO25A) could reflect a transitional stage in the wound healing process, wherein fibroblasts shift from a pro-angiogenic, proliferative phase to one focused on matrix deposition and remodeling. Taken together, these changes support the idea that mTOR signaling in fibroblasts during wound healing is finely tuned, enabling metabolic adaptation, controlled biosynthesis, and appropriate stress responses. This knowledge could inform targeted modulation of mTOR activity in fibroblast-based therapies for impaired wound healing, such as in diabetic ulcers or fibrotic conditions.

Dermal fibroblasts play a central role in the wound healing process, especially in inflammation, proliferation, and tissue remodeling phases, by producing ECM components, secreting cytokines, and modulating immune and angiogenic signals. The transcriptional activity of fibroblasts during wound repair reflects their functional state and determines the quality and efficiency of healing. hDFs on MdEV-SA scaffolds exhibited a distinct gene expression profile consistent with an active early to-intermediate wound healing phase, characterized by pro-inflammatory activation and selective ECM remodeling (Figure 8C). This gene expression pattern reflects a wound healing environment dominated by early myofibroblast activation and matrix restructuring with selective suppression of excessive inflammation, angiogenesis, and ECM deposition. The upregulation of ACTA2 and MMP2, alongside IL-6, indicates a fibroblast phenotype aligned with tissue contraction, matrix remodeling, and paracrine signaling to coordinate immune and epithelial responses. Conversely, the broad downregulation of collagens, matrix-degrading enzymes (MMP7/9), and immune mediators (CXCL11, IL2, IL4, IL-10) suggests that the system is moving away from an inflammatory and proliferative state, toward the resolution phase of repair. The reduction in HGF, EGF, and ITGA4 may further indicate a decrease in epithelial and vascular activation, consistent with the maturation of the granulation tissue. Altogether, this transcriptional signature portrays fibroblasts that are transitioning from a reactive to a remodeling state, where controlled matrix degradation, wound contraction, and partial immune resolution shape the final stages of healing. This profile could serve as a valuable reference for evaluating wound therapies or regenerative biomaterials.

#### 3.7.2 Gene Expression on MdEV-SA/HAP Scaffolds

The AMP-activated protein kinase (AMPK) pathway is a central regulator of cellular energy homeostasis, responding to changes in intracellular ATP/AMP ratio by modulating metabolism, proliferation, and stress adaptation.^35^ In human MSCs, AMPK plays a key role in maintaining stemness, regulating differentiation, and responding to inflammatory or hypoxic stress.^36^ The differential expression of key AMPK-related genes observed in MSCs reveals a complex pattern of partial activation and central inhibition, which may reflect adaptive metabolic rewiring (Figure 9A). The combined gene expression data suggest a unique MSC metabolic state characterized by peripheral activation of catabolic responses (ADRA—adrenergic receptor α and MLYCD—malonyl-CoA decarboxylase) in the context of central suppression of AMPK signaling (PRKA—AMPK catalytic subunit α, STRADB—STE-related adaptor protein β, and ACACA—acetyl-CoA carboxylase α). This dissociation may reflect an adaptive response to environmental stress or a functional reprogramming aimed at energy optimization.

**Figure 9:**
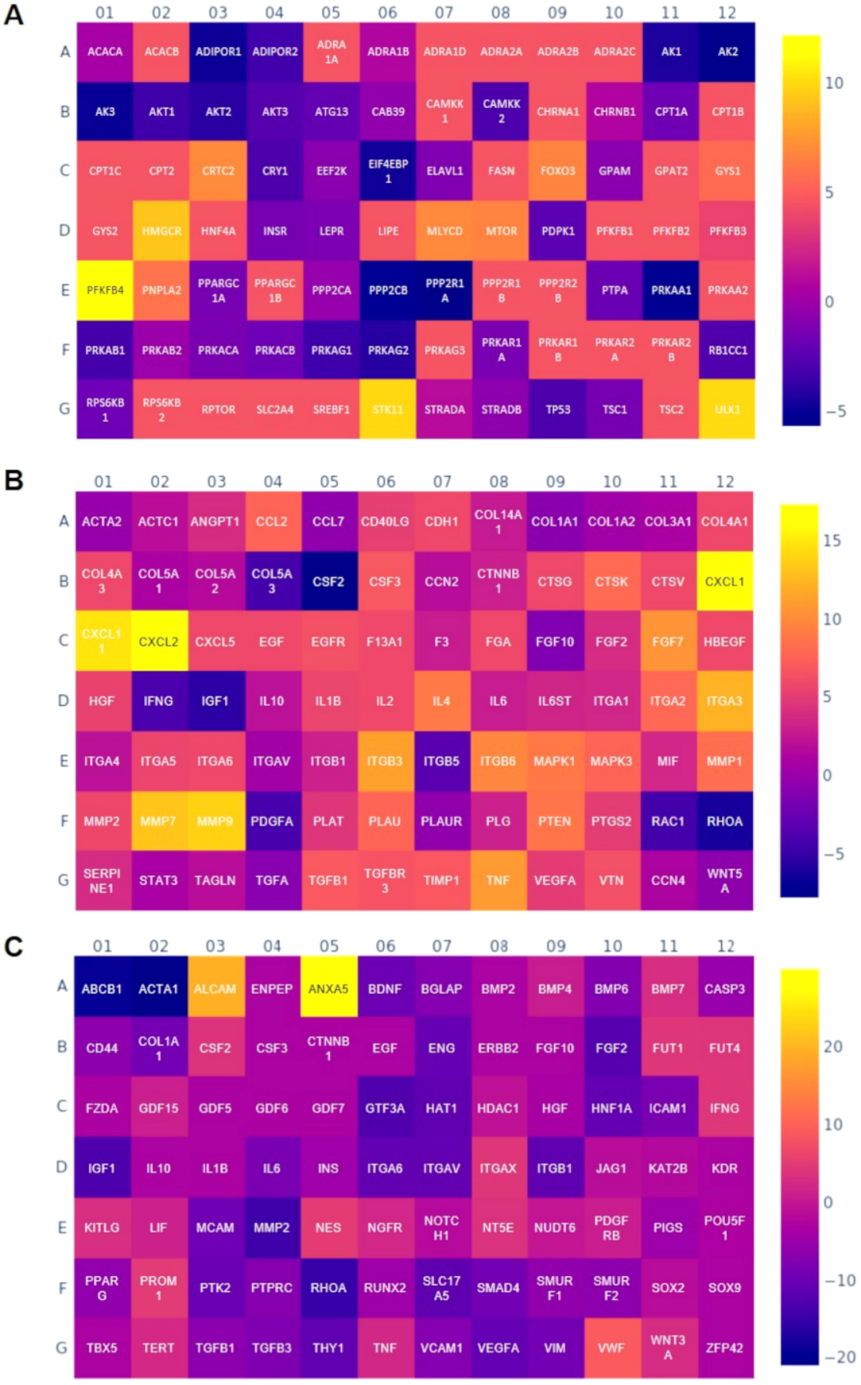
Gene expression profile of human mesenchymal stem cells on SA/HAP scaffolds loaded with microalgae-EVs compared with SA/HAP scaffolds. (A) AMPK-AKT signaling; (B) wound healing signaling; and (C) stem cell signaling. Heat map Log(2) fold change.

The molecular response of MSCs during wound healing is tightly orchestrated and involves genes associated with ECM remodeling, angiogenesis, inflammation, immune modulation, and tissue regeneration.^37^ The gene expression profile analyzed here shows a robust upregulation of key pro-regenerative factors, alongside the downregulation of specific structural and signaling elements, suggesting a pro-healing, antifibrotic MSC phenotype (Figure 9B). Specifically, the upregulation of key growth factors such as VEGF, angiopoietin (ANGPT), hepatocyte growth factor (HGF), and epidermal growth factor (EGF) reflects a strong commitment to neovascularization and epithelial regeneration. These molecular signals are likely to contribute to accelerated wound closure and a reduced risk of chronic wound persistence. Furthermore, the cytokine profile suggests a well-orchestrated inflammatory response, with the presence of both early pro-inflammatory markers (IL1 and IL-6) and anti-inflammatory mediators (IL-10, IL4, and TGF β). This balance indicates a controlled immune response that may promote regenerative healing, support M2 macrophage polarization, and limit the progression to chronic inflammation. In terms of ECM remodeling, the observed downregulation of fibrotic markers such as collagen type V (COL5) and RhoA, along with the moderated expression of ECM regulators including COL4 and COL14 and tissue inhibitors of metalloproteinases (TIMPs), suggests an environment conducive to matrix restructuring without excessive scar formation or fibrosis. Additionally, signaling pathways involving integrins and microtubule affinity-regulating kinases (MARK) point toward a MSC phenotype that is highly motile and interactive with ECM. This enhanced motility likely facilitates MSC homing, engraftment, and paracrine activity at the site of injury, thereby further supporting tissue repair and regeneration.

In SA/HAP scaffolds loaded with MdEVs compared, MSCs appear to retain stemness characteristics, modulate immune signaling, and suppress lineage-specific chondrogenic or adipogenic differentiation pathways in favor of osteogenic differentiation (Figure 9C). Several up-regulated genes are associated with the preservation of stem cell identity and multipotency. Markers such as PROM1 (CD133), NT5E (CD73), NES, TERT, NGFR, and LIF indicate a sustained undifferentiated state, while the presence of WNT3A and KITLG implies the activation of early developmental and regenerative signaling pathways. The upregulation of BMP7 and GDF15 supports a role in tissue repair and stress response rather than lineage-specific differentiation. Additionally, increased expression of immune-related genes such as CSF2, IFNG, and TNF, along with adhesion molecules such as ALCAM and ITGAX, suggests that the cells may adopt an immunomodulatory phenotype with enhanced communication with immune or endothelial cells. Conversely, there is a broad downregulation of genes associated with MSC differentiation and extracellular matrix remodeling. Genes required for chondrogenic differentiation such as SOX9, GDF5, GDF6, and TGFB family members are down-regulated, as are adipogenic regulators like PPARG and INS, suggesting a general inhibition of mesenchymal lineage commitment. Critical osteogenic markers, including RUNX2, COL1A1, BGLAP, BMP2, BMP6, and FGF2, are slightly expressed despite the osteoconductive nature of the HA component in the scaffold. Interestingly, the scaffold environment also appears to repress genes involved in fibrosis and inflammation. Markers such as MMP2, ACTA2, VIM, ICAM1, and RHOA, which are typically associated with fibrotic activity and cellular migration, show reduced levels of expression. Inflammatory and proliferative signals, including IL1B, IL-6, and CD44, and growth factors, such as EGF and HGF, are also diminished, pointing toward a quiescent or regulated immune state rather than an active inflammatory response.

Overall, this gene expression profile indicates that the SA-HAP scaffold supports the maintenance of MSC multipotency and immunomodulatory potential while suppressing differentiation and fibrotic signaling.

The gene expression results obtained in this study were further validated at the protein level by ELISA assays and directly compared with those of EV-free SA/HAP scaffolds. To ensure a homogeneous and comparable evaluation across experimental conditions, protein levels were not reported as absolute concentration but rather expressed as percentage variation relative to the control condition, defined as SA/HAP scaffolds without MdEVs (Figure 10).

**Figure 10:**
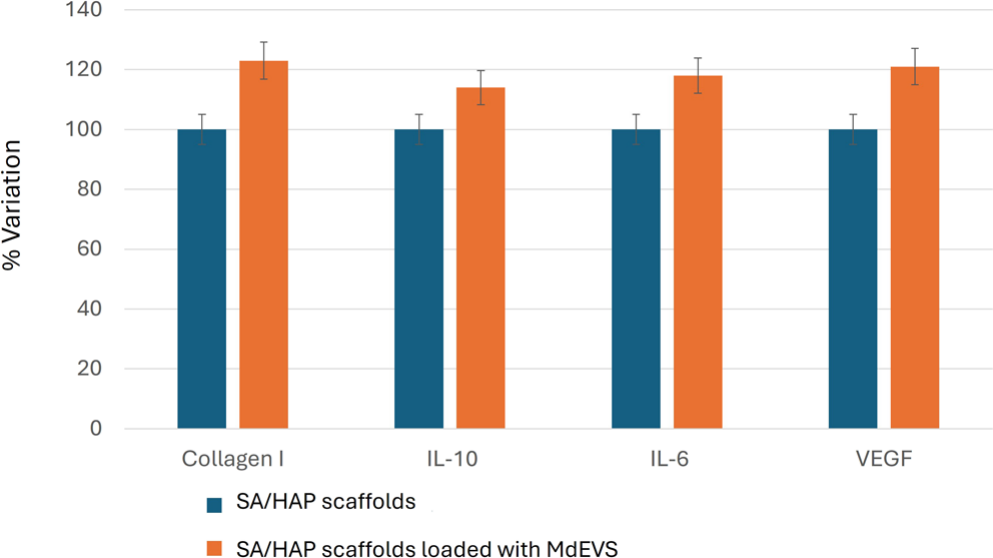
Protein-level validation of VEGF, IL-6, IL-10, and collagen I of human mesenchymal stem cells on SA/HAP scaffolds loaded with microalgae-EVs compared with SA/HAP scaffolds. Results are reported in terms of % variation.

## 4 Conclusions

This study presents a bioinspired, in vitro approach to promote the concurrent regeneration of skin and bone tissues using 3D printed sodium alginate (SA)-based hydrogels enriched with extracellular vesicles derived from *E. oleoabundans*, a green microalga (MdEVs). The integration of MdEVs within SA and alginate/hydroxyapatite (SA/HAP) matrices created a multifunctional and cytocompatible platform capable of activating distinct cellular programs relevant to both cutaneous and osseous healing, respectively. The transcriptional responses observed in both skin- and bone-relevant cellular models must be interpreted in the context of the physicochemical properties of the underlying scaffolds, as the biological outcomes reported here emerge from a finely orchestrated interplay between material-derived cues and MdEV-mediated biochemical signaling. In particular, the pro-osteogenic tendency exhibited by human mesenchymal stem cells (hMSCs) cultured on MdEV-loaded SA/HAP scaffolds, as evidenced by the gene expression profile shown in Figure 9, is likely driven by the convergence of osteoconductive and mechanotransductive stimuli provided by the HAP phase with the paracrine signaling activity of MdEVs. The incorporation of HAP within the SA matrix increases local stiffness, provides a calcium- and phosphate-rich microenvironment, and introduces mineral topographical features that are known to activate integrin-mediated adhesion, cytoskeletal tension, and downstream mechanosensitive pathways that bias mesenchymal stem cells toward osteogenic lineage commitment. Within this primed mechanical and chemical niche, MdEVs, successfully released from the scaffolds (Figure S1), appear to function as biochemical amplifiers, reinforcing the expression of osteoinductive and pro-angiogenic genes while concurrently suppressing fibrotic markers, thereby promoting a regenerative rather than pathological differentiation trajectory. Notably, this transcriptional activation occurs in the absence of overt terminal differentiation, suggesting that the combined material- and vesicle-derived signals maintain hMSCs in a poised, pro-regenerative state that is advantageous for controlled bone repair. Conversely, in the softer, highly hydrated SA-only matrix designed to recapitulate the mechanical compliance of native dermal tissue, MdEVs preferentially supported human dermal fibroblast (hDFs) proliferation and induced gene networks associated with extracellular matrix (ECM) remodelling, angiogenesis, and AKT/mTOR signaling, underscoring the context-dependent nature of MdEV bioactivity. These observations collectively indicate that MdEVs do not act as generic stimulatory agents, but rather as adaptable signaling entities whose biological effects are shaped by the surrounding material environment. Such synergy between “material signals,” including stiffness, mineral content, and osteoconductive properties, and “biochemical signals” conveyed by MdEV cargo likely underpins the multifunctional behavior of the platform, enabling the selective activation of distinct cellular programs relevant to skin and bone regeneration within a unified biofabricated system. The biological effects observed in this study indicate that MdEVs primarily act by potentiating the regenerative competence of hMSCs, rather than directly inducing functional responses in lineage-committed or terminally differentiated cell types. In MdEV-loaded SA/HAP constructs, hMSCs exhibited a highly coordinated transcriptional profile characterized by the preservation of stemness-associated traits, the modulation of immune-related signaling pathways, and the selective repression of alternative differentiation programs, including chondrogenic and adipogenic lineages, concomitant with a bias toward osteogenic commitment. This gene expression landscape reflects an enhanced regenerative readiness and plasticity of hMSCs, positioning them in a poised state that is favorable for subsequent tissue repair processes rather than for immediate terminal differentiation. Importantly, our in vitro data demonstrate that endothelial cells, macrophages, and mature osteoblasts do not display measurable recognition or internalization of microalgae-derived extracellular vesicles (data not shown). As a consequence, the regenerative effects associated with MdEV exposure are most plausibly mediated indirectly through MSC-driven mechanisms. hMSCs are well recognized as central coordinators of tissue regeneration, exerting their effects via paracrine signaling, immunomodulation, and lineage-guided matrix remodeling, thereby influencing angiogenesis, inflammatory resolution, and mineralized tissue formation. Within this hierarchical regenerative framework, MdEVs function upstream by modulating MSC behavior and amplifying their reparative signaling output, which, in turn, orchestrates downstream cellular and tissue-level responses. Accordingly, the transcriptional changes reported here should be interpreted as molecular indicators of MSC potentiation and regenerative directionality, representing early and biologically meaningful events that precede functional outcomes. While in vivo validation and functional assays involving secondary effector cell populations will be essential to fully elucidate the downstream consequences of this indirect mode of action, the present findings provide mechanistic insight into how microalgae-derived EVs enhance MSC intrinsic regenerative programs. This MSC-centered mechanism underscores the potential of MdEV-enriched biomaterials as advanced regenerative platforms capable of harnessing endogenous repair pathways through indirect but biologically coherent signaling strategies.These findings demonstrate that MdEVs can act as versatile paracrine modulators, adapting their signaling activity according to the cellular microenvironment and scaffold composition. The bioactive role of MdEVs in skin models may be attributed to specific lipid mediators and small noncoding RNAs previously identified in vesicles from marine and plant sources, known to modulate macrophage polarization, fibroblast proliferation, and keratinocyte migration, all crucial processes in wound closure and dermal architecture restoration.^38^

Beyond their biological activity, the use of algae-derived vesicles introduces a sustainable, ethically neutral, and highly scalable source of bioactive molecules for regenerative applications. Unlike mammalian EVs, MdEVs avoid the limitations associated with donor variability, immunogenicity, and ethical concerns while offering a renewable production system compatible with large-scale and cost-effective biomanufacturing. Although this study is limited to in vitro validation, the results provide solid proof of concept that environmentally sustainable EV-based systems can be engineered into 3D printed, tissue-specific scaffolds with potential translational value in the treatment of chronic skin ulcers, diabetic wounds, or complex defects involving both soft and hard-tissue loss. Future studies will focus on evaluating MdEV behavior within complex in vivo microenvironments, such as inflammatory and hypoxic conditions, and on optimizing EV loading and release kinetics to further enhance therapeutic efficacy. Overall, the proposed MdEV-loaded alginate platform represents an innovative, dual-action regenerative system that unites sustainable biotechnology with advanced bioprinting strategies to address clinically challenging wounds requiring simultaneous bone and skin repair.

## Supplementary Material



## Notes

Additional experimental details, materials, and methods on the quantification of MdEVs released from scaffolds based on the total protein content (μg × mL^–1^) (PDF)
