# Supplementary material for: Microalgae-Derived Extracellular Vesicle-Loaded 3D Alginate Hydrogels Promote In Vitro Skin and Bone Repair through Dual Fibroblast and Mesenchymal Stem Cell Modulation
Source: ACS Appl Bio Mater. 2026 Jan 3;9(2):1167–82. doi: 10.1021/acsabm.5c02229 (PMC12820966; doi:10.1021/acsabm.5c02229)
Supplement: Supplementary file 1 [file mt5c02229_si_001.pdf]

Supporting Information

Microalgae-derived extracellular vesicle-loaded 3D alginate hydrogels promote in vitro skin and bone repair through dual fibroblast and mesenchymal stem cell modulation

Noemi De Cesare [1,2§], Luna Ardondi [2,§], Tommaso Pusceddu [2], Lucia Sileo [2], Maria Pia Cavaleri [2], Ilaria Vitali [2], Francesco Grassi [4], Brunella Grigolo [4], Giuseppe Pezzotti [5], Ugo D'Amora [1,], Letizia Ferroni [3,+,*], Alfredo Ronca [1,6,+] and Barbara Zavan [2,3,+,*]*

[1] Institute of Polymers, Composites and Biomaterials - National Research Council (IPCB-CNR), Naples, 80125, Italy

[2] Department of Medical Sciences, University of Ferrara, 44121 Ferrara, Italy

[3] Maria Cecilia Hospital, GVM Care and Research, Cotignola, 48033, Italy; lferroni@gvmnet.it

[4] Laboratorio RAMSES, IRCCS Istituto Ortopedico Rizzoli, Bologna, Italy

[5] Biomedical Engineering Center, Kansai Medical University, 1-9-11 Shin-machi, Hirakata, Osaka 573-1191, Japan

[6] Institute of Polymers, Composites and Biomaterials - National Research Council (IPCB-CNR), Lecco, 23900, Italy;

Keywords: microalgae-derived extracellular vesicles; diabetic ulcer care; sodium alginate; hydroxyapatite; 3D printed hydrogel

[*] Corresponding authors: Ugo D'Amora (ugo.damora@cnr.it), Barbara Zavan (barbara.zavan@unife.it) and Letizia Ferroni (lferroni@gvmnet.it).

[§] These authors contributed equally to the work

[+] Co-last authors

Release Study

Scaffolds were soaked with MdEVs for 20 min to allow complete rehydration of the biomaterial. The MdEV release profile was assessed by incubating the functionalized scaffolds in a 24-well plate containing serum-free culture medium. Protein content was quantified using a BCA Protein Assay (Thermo Fisher Scientific) according to the manufacturer's instructions at baseline (T0) and after 1, 6, 12, and 24 h, in both the scaffolds and the culture medium. Data are presented as the mean ± SD of six replicates.

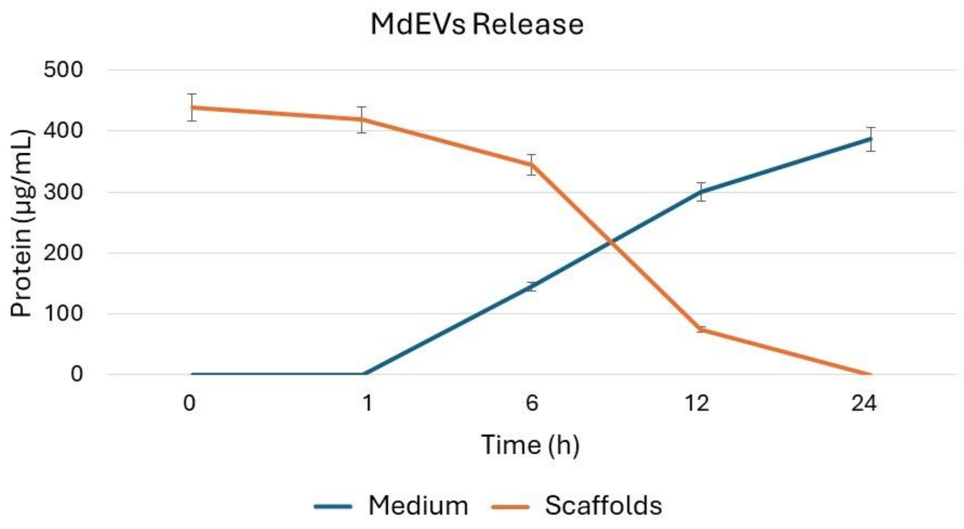

Figure S1. MdEVs release from scaffolds in terms protein express as μg×mL^{-1}.

Quantitative analysis of MdEV adsorption demonstrated efficient vesicle loading into both alginate/hydroxyapatite (SA/HAP) hydrogels following the 20 min soaking procedure.

BCA assessment of the post-loading supernatants revealed that a substantial fraction of MdEV-associated protein was retained within the hydrogel matrices, confirming the effectiveness of the passive adsorption strategy. When incubated under physiological conditions, SA/HAP scaffolds displayed a sustained, material-dependent MdEV release profile characterized by an initial release phase during the early time points, followed by a more gradual and controlled release over time. Collectively, these findings demonstrate that hydrogel composition critically governs MdEV loading and release behavior, providing enhanced vesicle stabilization and sustained delivery, thereby offering a more controlled and potentially therapeutically relevant MdEV presentation profile.
